# Inhibitor of DNA binding‐1 is a key regulator of cancer cell vasculogenic mimicry

**DOI:** 10.1002/1878-0261.70027

**Published:** 2025-03-21

**Authors:** Emma J. Thompson, Emma L. Dorward, Kristyn Jurrius, Nathalie Nataren, Markus Tondl, Kay K. Myo Min, Michaelia P. Cockshell, Anahita Fouladzadeh, John Toubia, Mark DeNichilo, Delphine Merino, Claudine S. Bonder

**Affiliations:** ^1^ Centre for Cancer Biology University of South Australia and SA Pathology Adelaide Australia; ^2^ ACRF Cancer Genomics Facility, Centre for Cancer Biology University of South Australia and SA Pathology Adelaide Australia; ^3^ Olivia Newton John Cancer Research Institute Melbourne Australia; ^4^ School of Cancer Medicine La Trobe University Bundoora Australia; ^5^ Department of Medical Biology, The Faculty of Medicine, Dentistry and Health Science The University of Melbourne Australia; ^6^ Immunology Division The Walter and Eliza Hall Institute of Medical Research Parkville Australia; ^7^ Adelaide Medical School University of Adelaide Australia

**Keywords:** breast cancer, ID1, pancreatic cancer, tumour vasculature, vasculogenic mimicry

## Abstract

Solid tumours routinely access the blood supply by promoting endothelium‐dependent angiogenesis; but tumour vasculature can also be formed by cancer cells themselves via vasculogenic mimicry (VM). Investigation of the gene expression profile during the early stages of VM formation by MDA‐MB‐231‐LM2 breast cancer cells identified the transcriptional regulator inhibitor of DNA binding 1 (*ID1*) to be elevated ~ 10‐fold within the first 2 hours. This role for ID1 in promoting VM was supported by *ID1* genetic knockdown or chemical inhibition interrupting VM formation by MDA‐MB‐231‐LM2 (breast) and BxPC‐3 (pancreatic) cancer cells. More specifically, reducing ID1 lowered cancer cell expression of endothelial cell genes (e.g. *CDH5*, *TIE2*) and production of pro‐angiogenic proteins (e.g. VEGF, CD31, MMP9 and IL‐8). *In silico* analysis of MDA‐MB‐231 cells engrafted into mice identified elevated *ID1* expression in cancer cells that had metastasised to the lungs or liver, and an enrichment of pro‐angiogenic genes. Additionally, *Id1* knockdown in 4T1.13 murine breast cancer cells demonstrated reduced tumour growth and metastasis *in vivo*. Taken together, this study further implicates ID1 in a vascular program within cancer cells that supports disease progression.

## Graphical abstract

Elevated expression of transcriptional regulator inhibitor of DNA binding 1 (ID1) promoted cancer cell‐mediated vasculogenic mimicry (VM) through regulation of pro‐angiogenic and pro‐cancerous genes (e.g. VE‐cadherin (*CDH5*), *TIE2*, *MMP9*, *DKK1*). Higher *ID1* expression also increased metastases to the lung and the liver. Genetic manipulation, or ID1 reduction with pan‐ID inhibitor AGX‐51,s was able to attenuate these effects. Made with Biorender.com.

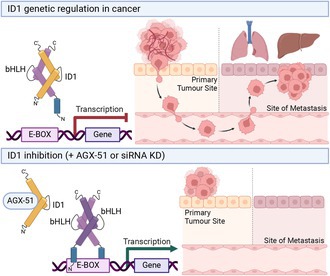

**Abbreviations**
2D: 2‐dimensional
3D: 3‐dimensional
BSA: bovine serum albumin
CPM: counts per million
CYCA: cyclophilin A
DGE: differential gene expression
DKK1: Dickkopf‐1
DMEM: Dulbecco's modified Eagle medium
DMSO: dimethylsulphoxide
EC: endothelial cell
ECGS: endothelial cell growth supplement
ECM: extracellular matrix
EMMPRIN: extracellular matrix metalloproteinase inducer
EMT: epithelial‐to‐mesenchymal transition
FBS: fetal bovine serum
GAPDH: glyceraldehyde‐3‐phosphate dehydrogenase
GO: gene ontology
HLH: helix–loop–helix
HUVE: human umbilical vein endothelial
ID1: inhibitor of DNA binding
IHC: immunohistochemistry
IL: interleukin
LeGO: lentiviral gene ontology
LucGFP: luciferase green fluorescent protein
mAb: monoclonal antibody
MES: 2‐(*N*‐morpholino)ethanesulfonic acid
miRNA: micro ribonucleic acid
MMP: matrix metalloprotease
NSG: NOD scid gamma
PAI‐1: plasminogen activator inhibitor‐1
PAS: periodic acid schiff
PBS: phosphate buffered saline
PDAC: pancreatic ductal adenocarcinoma
PDGF: platelet‐derived growth factor
RPMI: Roswell Park Memorial Institute
siRNA: small interfering ribonucleic acid
STAR: Spliced Transcripts Alignment to a Reference
TCP: tissue culture plastic
TMM: trimmed mean of M‐values
TNBC: triple negative breast cancer
TrHBMEC: transformed human bone marrow endothelial cells
VEGF: vascular endothelial growth factor
VM: vasculogenic mimicry

## 1 Introduction

Cancer remains a leading cause of death worldwide, with particular subtypes of cancer (e.g. triple negative breast cancer (TNBC) and pancreatic ductal adenocarcinoma (PDAC)) proving extremely difficult to treat [1, 2]. Cancer cell metastasis to vital organs (such as the lungs and liver) is frequently the final and fatal step of the progression of solid malignancies and is dependent on tumour vascularisation [3]. Tumours can develop a blood supply not only by promoting angiogenesis (endothelial cell (EC)‐lined vasculature) but also by cancer cells themselves that can form vessel‐like structures in a process known as vasculogenic mimicry (VM) [4, 5, 6]. While abundant VM has been associated with increased invasion, high tumour grade, metastasis, and overall poor survival for several cancer types [7, 8], VM‐specific markers are lacking, and the molecular mechanisms that underpin VM formation remain poorly understood [9].

In the absence of VM‐specific markers, these fluid‐conducting intratumoural structures are measured by immunohistochemistry (IHC) with strong positive staining for Periodic acid‐Schiff (PAS) that are devoid of EC markers (e.g. CD31, CD34, VE‐cadherin) [9, 10]. From tumour‐based data, the average incidence of VM is ~ 30% with the overwhelming consensus that VM is frequently observed in highly aggressive tumours and correlates with poor prognosis [7, 8, 9]. At first, the contribution of VM to solid malignancies was presumed to be restricted to oxygen supply, nutrient transport, and the elimination of cell waste [4, 5]. More recently, evidence suggests that VM‐competent cancer cells produce anti‐coagulation proteins [11] and VM structures allow the transport of cells (red blood cells (RBCs), leukocytes and platelets) for active participation in tumour immunity [12, 13, 14]. Due to the compelling evidence that VM is involved in resistance to conventional therapies [15], a better understanding of VM is warranted to foster the development of new targeted (and effective) approaches to stop cancer progression.

Previous studies to decipher the mechanisms involved in VM formation include gene expression analysis of melanoma cells and ECs cultured in three‐dimensional (3D) gels of polymerised collagen for 14 days, or Matrigel for 2 days, followed by laser‐capture microdissection of the vascular networks for gene expression analysis by microarray [16]. Results provided strong evidence for cancer cell plasticity by the melanoma cells and a dominant pro‐angiogenic gene profile [16]. Consistent with this, additional molecular pathways supporting VM include hypoxia, components of the extracellular matrix, the PI3K‐Akt pathway, the Wnt signalling pathway, and epithelial‐mesenchymal transition (EMT)‐related proteins [9, 17]. Notably, the early regulatory mechanisms that underpin VM formation remain poorly understood.

This study aims to identify genes of early VM regulatory events. To address this, the human TNBC cell line MDA‐MB‐231‐LM2 [18] was cultured in either two dimensions (2D, tissue culture flasks) or in 3D as VM structures on Matrigel and harvested at early time points of VM formation (i.e. 2 and 6 h). The structures were then analysed by bulk RNA sequencing and comparative gene expression analysis. From this, we report that among the genes significantly upregulated within 2 h of seeding the VM assay, most notable was the transcription factor inhibitor of DNA binding 1 (ID1). Known to regulate biological processes such as cell differentiation, proliferation, migration, and angiogenesis [19], *ID1* expression by cancer cells is associated with poor patient prognosis [20, 21]. However, the role of ID1 in VM formation is largely unknown. Herein, inhibitor studies in TNBC and PDAC cells identified a previously underappreciated role for ID1 in the formation of VM and the production of pro‐cancerous cytokines, growth factors and signalling molecules. Moreover, gene ontology of ID1‐associated TNBC metastasis *in vivo* indicated a pro‐angiogenic phenotype of the MDA‐MB‐231 cells which is consistent with VM capability and disease progression.

## 2 Materials and methods

### 2.1 Cell lines and culture

MDA‐MB‐231‐LM2 breast cancer cells (derivative of CVCL_0062, kindly provided by J. Massagué (Sloan‐Kettering Institute for Cancer Research, New York, NY, USA)) were cultured in DMEM (Gibco, Thermo Fisher Scientific, Waltham, MA, USA) supplemented with 10% FBS (HyClone, Logan, UT, USA). AsPC‐1 (CVCL_0152) and BxPC‐3 (CVCL_0186) pancreatic cancer cells (purchased from CellBank Australia (Children's Medical Research Institute, Westmead, Sydney, NSW, Australia)) were cultured in RPMI (Gibco) supplemented with 10% FBS. . Murine 4T1.13 (triple negative breast cancer cell line) was kindly gifted by R. L. Anderson (Olivia Newton‐John Cancer Research Institute, Melbourne, Victoria, Australia) and lentivirally transduced to stably express luciferase and GFP for use in *in vivo* experiments. Cells were maintained in DMEM (Gibco) supplemented with 10% FBS (Hyclone). All cell lines were confirmed mycoplasma negative (MycoAlert, Lonza, Basel, Switzerland), were authenticated by short tandem repeat (STR) profiling, and maintained in culture at 37 °C, 5% CO_2_.

### 2.2 VM formation and angiogenesis assay

*In vitro* VM assays of cancer cells were performed using Growth Factor Reduced Matrigel (Corning™, Corning, NY, USA) in the Angiogenesis μ‐slides (Ibidi, Gräfelfing, Germany). Briefly, 1.75–3.5 × 10^4^ cancer cells were seeded onto a layer of Matrigel and images were captured periodically over 24 h using an inverted brightfield microscope (EVOS XL; Life Technologies, Carlsbad, CA, USA) or disk confocal live microscopy (CV100; Olympus, Tokyo, Japan). Still images were merged using Adobe Photoshop and vascular‐like structures were manually counted using the imagej software Cell Counter plugin (1.48pv; NIH, Bethesda, MD, USA). Vascular‐like structures were defined as multi‐cellular elongated arrangements tightly aligned and extending between collections of cells as previously described [22].

Using similar experiments (with details listed below), cells were harvested for gene expression analysis, pre‐treated with ID1‐inhibiting reagents, or culture supernatants collected for protein analysis.

### 2.3 Bulk RNA sequencing

MDA‐MB‐231‐LM2 cells were cultured under normoxic conditions (ambient 21% O_2_ with 5% CO_2_), either as 2D on tissue culture plastic (TCP) (1.5 × 10^5^ cells/well in an uncoated 24‐well plate, 3 wells combined for each replicate) or as 3D Matrigel (VM) cultures (1.5 × 10^5^ cells/well in 24‐well plate coated with 150 μL Matrigel (Corning™) diluted 1 : 1 in PBS, 3 wells combined for each replicate) and cells were harvested over a time course of 2 and 6 h. All samples were prepared in triplicate.

To harvest the 2D TCP cells, flasks were washed with PBS, then lifted from the TCP with 0.01 m EDTA in PBS, 500 μL of complete media was added to the well. The cell suspension was then collected and centrifuged for 5 min at 300 ***g***. To harvest the 3D VM cells, the plate was placed on ice, and culture medium was removed prior to 300 μL of neat Dispase (Falcon, Corning, NY, USA) added to dissolve the Matrigel layer for 15 min, followed by 500 μL of cold PBS being added, and the entire contents of the well collected and centrifuged for 5 min at 300g. The pelleted cells (both 2D and 3D cells) were washed with 1 mL cold PBS prior to the cells being immediately prepared for RNA sequencing. RNA was isolated using the mirVana™ miRNA isolation kit (Ambion™, Life Technologies, Carlsbad, CA, USA) as per the manufacturer's instructions.

RNA sequencing was outsourced to the ACRF Cancer Genomics Facility (Adelaide, SA, Australia). RNA integrity was established with a Bioanalyzer 2100 (Agilent Technologies, Santa Clara, CA, USA) trace. Poly(A) enriched mRNA libraries were constructed using the KAPA RNA HyperPrep kit (KK8541) and sequenced in triplicate biological replicates on two sequential runs of the Illumina NextSeq 500 platform using the stranded single‐end protocol with a read length of 75 bp. Raw reads were adaptor trimmed and filtered for short sequences using cutadapt v1.8.1 [23], setting the minimum‐length option to 18, error rate 0.2, and overlap 5. The resulting fastq files were analysed, and quality checked using the fastqc program (http://www.bioinformatics.babraham.ac.uk/projects/fastqc). The trimmed, quality‐checked fastq files for matched samples derived from the two sequential runs were subsequently merged. The merged files, averaging 49.7 million reads per sample, were mapped against the human reference genome (hg19) using the star (Spliced Transcripts Alignment to a Reference) algorithm [24] (version 2.5.3a with default parameters and ‐‐chimSegmentMin 20, ‐‐quantMode GeneCounts) returning an average unique alignment rate of 87.9%. Differential expression analysis was evaluated from Trimmed Mean of *M*‐values (TMM) normalised gene counts using r (version 3.2.3) and edger [25] (version 3.3) following protocols as described in Refs [26, 27]. Functional analyses were performed using the topgo package in r. Alignments were visualised and interrogated using the integrative genomics viewer v2.4.6 [28].

### 2.4 ID1 knockdown

#### 2.4.1 Small interfering RNA (siRNA) transient knockdown of *ID1*

Transient silencing of human *ID1* expression was achieved by treating cells with trilencer‐27 siRNA duplexes of three different sequences targeting human *ID1* (siRNA Oligo Duplex (Locus ID 3397; OriGene, Rockville, MD, USA)). Duplexes were reconstituted at 20 μm as per the manufacturer's instructions. Cells were then treated with the transfection complex (siRNA stock at 20 μm and Lipofectamine® RNAiMAX (Invitrogen, Waltham, MA, USA) diluted in Opti‐MEM® reduced serum medium (Gibco)) with a final concentration of 10 nm of *ID1*‐targeting siRNA duplexes. For control, cells were also treated with 10 nm of the universal non‐silencing siRNA duplex (Origene). Knockdown efficiency was routinely assessed via western blot at 48–72 h post‐transfection.

#### 2.4.2 Inhibitor AGX‐51

AGX‐51 (MedChemExpress, Monmouth Junction, NY, USA) was reconstituted to 10 mm in DMSO as per the manufacturer's instructions, and cells were treated with 0–40 μm for 24 h prior to use in assays. ID1 protein levels were assessed via western blot.

### 2.5 Western blot

Cells were lysed using RIPA or EB buffer containing cOmplete™ Protease Inhibitor cocktail and PhosSTOP™ (one tablet of each added to 10 mL of buffer; Roche, Basel, Switzerland). Lysates were clarified via centrifugation at 6700 ***g*** for 15 min at 4 °C prior to heating to 90 °C for 5 min in SDS‐based reducing sample buffer, and samples were run in precast 4–12% Bis‐Tris gels (Criterion XT; BioRad, Hercules, CA, USA) using a MES‐based running buffer. Proteins were transferred onto a nitrocellulose membrane (PALL Life Sciences, Port Washington, NY, USA); the membrane was blocked with 5% BSA overnight at 4 °C and probed with antibodies to ID1 (mouse mAb, 1 μg·mL^−1^ (1 : 200), sc‐133104; Santa Cruz, Dallas, TX, USA), total actin (mouse mAb, 1 : 3000, MAB1501; Sigma‐Aldrich, St Louis, MO, USA) or GADPH (rabbit mAb, 1 : 3000, D16H11; Cell Signalling, Danvers, MA, USA), visualised using goat anti‐mouse (800CW, 1 : 10 000) or goat anti‐rabbit (680IR, 1 : 10 000) antibodies from Li‐COR Biosciences (Lincoln, NE, USA) on the LI‐COR Odyssey. For beta‐actin staining, the membrane was probed with anti‐β‐Actin‐peroxidase (mouse mAb, 1 : 500, A3854; Sigma‐Aldrich), then immersed in ECL western blotting reagents (RPN2106; Cytiva, Marlborough, MA, USA). Signal was captured with the Fujifilm LAS 4000 imager (Minato City, Tokyo, Japan). Raw data was exported to Original Image By Colour TIFF using the multi gauge v3.0 software (FUJIFILM, Tokyo, Japan). TIFF files were imported into image studio lite v5.2 (LI‐COR Biosciences) for densitometry analysis. Protein levels were normalised for loading controls within respective lanes (e.g. GAPDH or beta‐actin) and normalised to experimental controls for replicate experiments.

### 2.6 Cytokine array

Cells were treated with 20 μm AGX‐51 (or equal volume DMSO) for 24 h prior to seeding into the VM‐tube formation assay. After a further 24 h, the supernatant was collected and added to the Human XL Cytokine Array (R&D Systems, Minneapolis, MN, USA) as per the manufacturer's instructions. Membranes were imaged using the Chemidoc Touch Imaging system (BioRad). Densitometry analysis was performed using imagequant tl v8.1.0.0 (GE, Boston, MA, USA).

### 2.7 Gene expression and regulatory motif determination

Cells were treated with 20 μm of AGX‐51 (or equal volume DMSO (ctl)) for 24 h prior to seeding onto TCP or Matrigel. After 24 h, cells were harvested by Dispase digestion (as detailed above) followed by total RNA extraction using the RNeasy Micro Plus kit (Qiagen, Hilden, Germany) as per the manufacturer's instructions. Two micrograms of total RNA was converted into first‐strand cDNA using SuperScript III Reverse Transcriptase (Life Technologies, Carlsbad, CA, USA) as per manufacturer's instructions. QuantiTect SYBR Green (Qiagen) was used for qPCRs, and samples were analysed on Rotor‐Gene thermocyclers (Qiagen). Cycling parameters began with a 15 min hold at 95 °C, 45 cycles of 95 °C for 10 s, 55 °C for 20 s, and 72 °C for 30 s, followed by a melt phase. Oligonucleotide sequences are as follows: *CYCA* (cyclophilin A), 5′‐GGCAAATGCTGGACCCAACACAAA‐3′ and 5′‐CTAGGCATGGGAGGGAACAAGGAA‐3′; *CDH5* (VE‐Cadherin) 5′‐TGA CAA TGT CCA AAC CCA CTC A‐3′ and 5′‐TGA CAA CAG CGA GGT GTA AAG AC‐3′; *PECAM‐1* (CD31) 5′‐TCC TGT GAA ATA CCA ACC TGA AGA‐3′ and 5′‐GCT TGT TCC ACC TTC ATT TTC TG‐3′; *TIE2* 5′‐GTG CTG TTG GCC TTT CTG ATC‐3′ and 5′‐GAA GGC TTG GGC CAT TCT C‐3′; *VEGFR2* 5′‐ATC ACA CAA TTA AAG CGG GG‐3′ and 5′‐AAT CTG GGG TGG GAC ATA CA‐3′; *ID1* 5′‐TAGTCGATGACGTGCTGGAG‐3′ and 5′‐AAACGTGCTGCTCTACGACA‐3′; *MMP9* 5′‐CTATTTCTGCCAGGACCGCT‐3′ and 5′‐GTTGGTCCCAGTGGGGATTT‐3′; *IL‐8/CXCL8* 5′‐TTTTGCCAAGGAGTGCTAAAGA‐3′ and 5′‐AACCCTCTGCACCCAGTTTTC‐3′; *DKK‐1* 5′‐CCTTGAACTCGGTTCTCAATTCC‐3′ and 5′‐CAATGGTCTGGTACTTATTCCCG‐3′. Relative gene expression levels were calculated using comparative quantitation then normalised to the housekeeping gene *CYCA*.

### 2.8 Helix–loop–helix motif identification

For candidate genes identified in the Protein Profiler Array, a *cis*‐regulatory element was selected (USCS Genome Browser v438, hg38 [29]) based on H3K27Ac (open chromatin), H3K4me1 (enhancer) and H3K4me3 (promoter) histone modifications, as well as cCREs (ENCODE Candidate *cis*‐Regulatory Elements, and conservation data of 100 vertebrates [30]). MEME CHIP (SEA [31]) with default settings (HOCOMOCOv11 core) was used to identify enriched motifs with *P*‐value < 0.05. The sequence logo of basic helix–loop–helix motifs was taken from jaspar [32].

### 2.9 Analysis of differentially expressed genes and gene ontology

Sequencing data lodged under DOI: 10.5281/zenodo.10920838 from GSE193369 [33] was processed in R by employing the RNA‐Seq analysis platform degust v4.1.1 [34], with voom/limma, FDR < 0.05 and min Counts per Million (CPM) of 10. Gene Ontology (GO) was performed via the DAVID Functional Annotation Tool, using the GOTERM_BP_DIRECT gene set [35, 36] and results were filtered to show the top GO terms based on a false discovery rate ≤ 0.05. The output from the GO Biological Process gene set and GO term gene list filtering was performed in r (version 4.3.1) using r packages such as tidyverse, and plotting was performed using ggplot2.

### 2.10 Orthotopic TNBC xenograft model using *Id1* knockdown 4T1.13‐LucGFP cells

Trilencer‐27 siRNA duplexes of three different sequences targeting mouse *Id1* (siRNA Oligo Duplex (Locus ID 15901; OriGene)) were reconstituted at 20 μm as per the manufacturer's instructions. Transfection complex: siRNA stock solution (mixture of all 3 sequences) and Lipofectamine® RNAiMAX (Invitrogen) diluted in Opti‐MEM® reduced serum medium (Gibco). Transfection complexes were then added to a 6‐well plate of 4T1.13‐LucGFP cells (at ~ 20% confluency) in DMEM with 10% FBS at a final siRNA concentration of 50 nm. Cells were harvested at 72 h post‐transfection for injection into mice.

All animal experiments conducted in this study were approved by the Animal Ethics Committee of University of South Australia (Ethics approval #U27‐21 via Licence No. 155) and conform to the guidelines established by the ‘Australian Code for the Care and Use of Animals for Scientific Purposes’ issued by the Minister for Climate, Environment and Water under The Animal Welfare Act 1985. Mice were kept in specific pathogen‐free conditions in environmental temperature of 25 °C and a 12‐h light : dark cycle, with *ad libitum* access to standard rodent chow and water. All procedures and handling of mice was performed in accordance with approved ethical protocols.

5 × 10^5^ 4T1.13‐LucGFP cells (±siId1 or siNS), resuspended in 50 μL of a 1 : 1 PBS:Matrigel (Corning™) mixture, were orthotopically engrafted into the 4th mammary fat pad of 6–8‐week‐old, female BALB/c mice (purchased from Australian Bio Resources, Sydney, NSW, Australia). Tumour growth was assessed by bioluminescence imaging using the injection of 3 μg firefly d‐Luciferin solution (Cayman Chemical, Ann Arbor, MI, USA), visualised in the IVIS® Lumina S5 *In Vivo* Imaging System (Perkin Elmer, Waltham, MA, USA). At day 10, dissected tumours, lungs, leg bones, and livers were also harvested for assessment of bioluminescence.

### 2.11 Statistical analysis

One‐way ANOVA and Student's *t* tests were performed where suitable using graphpad prism software (GraphPad software Inc., San Diego, CA, USA). The variance between *ID1* counts per million expression data from primary tumour versus liver and lung was assessed using Bartlett's test statistic, and Welch's ANOVA for unequal variance with the Games‐Howell *post‐hoc* test was used to test the significance in expression between groups (confidence interval = 0.95) using r. Results with *P* < 0.05 were considered statistically significant.

## 3 Results

### 3.1 Gene expression profiling of vasculogenic mimicry (VM) by cancer cells

**Fig. 1:**
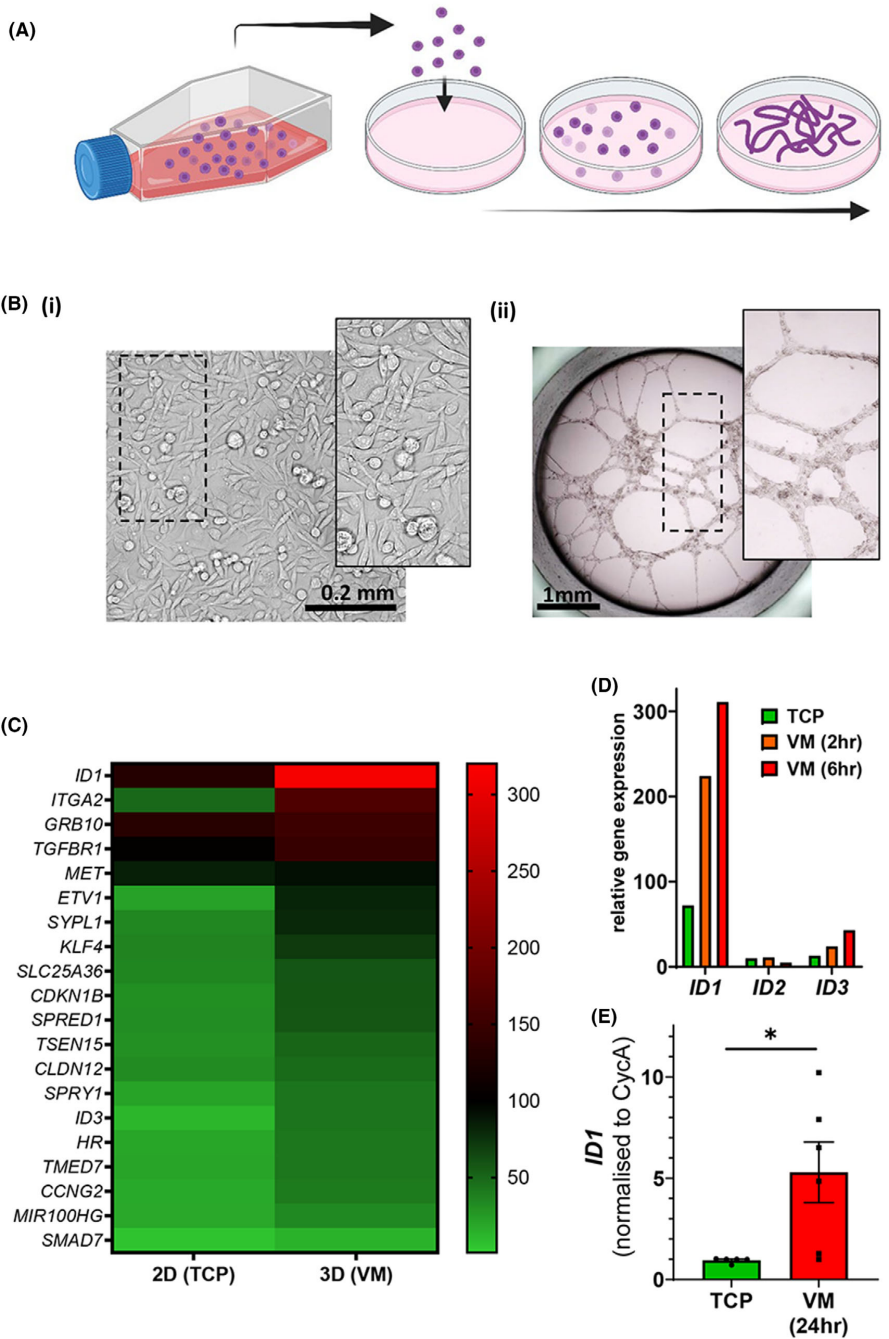
Vasculogenic mimicry by MDA‐MB‐231‐LM2 cells and gene expression profiling. (A) Schematic of cell culture in a flask (left) and single cell seeding into the Matrigel‐containing well to facilitate vascular structures. (B) Microscopy image of human breast cancer cells MDA‐MB‐231‐LM2 in (i) 2 dimensions (tissue culture plastic, TCP) and (ii) 3 dimensions (Matrigel vasculogenic mimicry assay, VM). (C) Heat map of RNAseq results depicting the top 20 differentially expressed genes in MDA‐MB‐231‐LM2 cells grown on TCP versus a VM assay. (D) Relative gene expression of *ID1, ID2* and *ID3* in MDA‐MB‐231‐LM2 cells grown on TCP versus a VM assay at 2 and 6 h time points. (E) Gene expression of *ID1* in MDA‐MB‐231‐LM2 cells cultured for 24 h on TCP or Matrigel. Data are expressed as mean ± SEM; TCP *n* = 5, VM *n* = 6, from three independent experiments. *P < 0.05 vs TCP, Welch's unpaired *t*‐test.

To determine alterations in gene expression during VM by cancer cells (Video S1), we performed whole transcriptome RNA sequencing on human TNBC MDA‐MB‐231‐LM2 cells cultured on tissue culture plastic (2‐dimensional (2D) on TCP) or in a Matrigel assay (3‐dimensional (3D) as VM) (Fig. 1A,B). Cells were harvested at either time zero (for 2D TCP) or at 2 or 6 h post‐seeding into the VM assay (for 3D VM) prior to the triplicate biological replicates being run on the Illumina NextSeq 500 platform using the standard single‐end protocol with a read length of 75 bp. The resulting fastq files were analysed, and quality checked with ~ 50 × 10^6^ reads per sample mapped against the human reference genome (hg19). Comparative expression analysis was undertaken (GSE272838) and identified over 25 genes that were differentially regulated during the process of VM (Fig. 1C). One gene of particular interest was inhibitor of DNA‐binding‐1 (*ID1*), with its expression increasing 4‐fold during the first 6 h of VM formation (Fig. 1D). In comparison, related family members *ID2* and *ID3* were more lowly expressed in cells on TCP and not elevated to the same extent in 3D culture (Fig. 1D) suggesting an important role for *ID1* in the formation of VM structures by TNBC cells. To note, a similar RNAseq analysis was performed on MDA‐MB‐231‐LM2 cells (2D and 3D) under hypoxic conditions (0.5% O_2_, 5% CO_2_ for 2 and 6 h) with further elevation of *ID1* observed across the groups (Fig. S1). The elevated *ID1* expression by MDA‐MB‐231‐LM2 cells during VM formation observed in the whole transcriptome RNAseq data was then replicated in independent qRT‐PCR gene expression analysis of MDA‐MB‐231‐LM2 cells grown in 2D on TCP or in 3D as VM for 24 h (Fig. 1E).

### 3.2 Knockdown of *ID1* inhibits VM formation by cancer cells

**Fig. 2:**
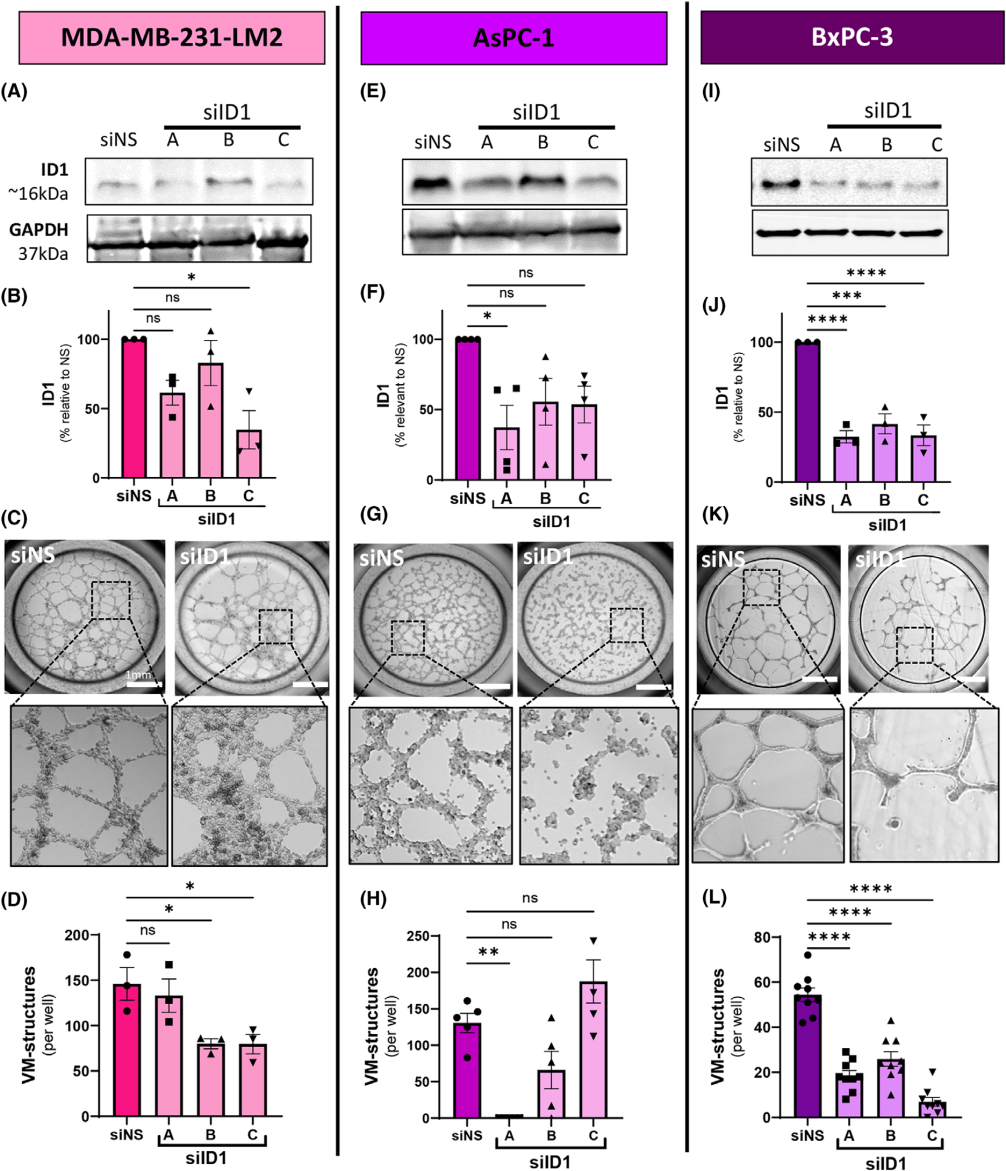
ID1‐targeting siRNA attenuates VM by cancer cells *in vitro*. (A, E, I) Representative images of ID1 protein expression in MDA‐MB‐231‐LM2, AsPC‐1, and BxPC‐3 cancer cells with siRNA for non‐silencing (siNS) or targeting *ID1* (siID1, constructs A–C) and loading control GAPDH. (B, F, J) Bar graphs of immunoblot densitometry quantified from experimental repeats, siID1 (constructs A–C) normalised to siNS controls. Data are expressed as mean ± SEM from *n* = 3 (MDA‐MB‐231‐LM2 and BxPC‐3) *n* = 4 (AsPC‐1) experiments. ns, not significant; *P < 0.05, ***P < 0.001, ****P < 0.0001 vs siNS, one‐way ANOVA. (C, G, K) Representative images of cancer cells undergoing vasculogenic mimicry (VM) with siNS or siID1 knockdown. For each cell line, the top image depicts VM with low magnification (scale bar = 1 mm) and the bottom image is a higher magnification of the selected region. (D, H, L) Quantitation of VM formation by cancer cells. Data are expressed as mean ± SEM from *n* = 3 (MDA‐MB‐231‐LM2, *n* = 5 (AsPC‐1), *n* = 9 (BxPC‐3)) individual experiments. ns, not significant; *P < 0.05, **P < 0.01, ****P < 0.0001 vs siNS, one‐way ANOVA.

To investigate the contribution of *ID1* on VM produced by MDA‐MB‐231‐LM2 cells, *ID1*‐targeting siRNAs were used to transiently reduce *ID1* gene and protein expression. Figure 2A,B show an immunoblot of endogenous ID1 protein levels in the TNBC cells and the corresponding reduction in expression following *ID1* knockdown with two of the three siRNA constructs. Next, we examined the VM capability of MDA‐MB‐231‐LM2 cells with or without *ID1* knockdown. Figure 2C,D show that the reduction in ID1 attenuates VM formation by these TNBC cells in two of the three siID1 groups. The noticeable low‐level expression of endogenous ID1 protein in MDA‐MB‐231‐LM2 cells, together with a modest reduction of ID1 and VM formation following siRNA knockdown, prompted a search for VM‐competent cancer cells with higher endogenous levels of ID1; i.e., human pancreatic cancer cell lines AsPC‐1 and BxPC‐3. Figure 2E–H illustrate that all three ID1‐targeting siRNAs reduce ID1 protein levels in the AsPC‐1 cells and a subsequent loss of VM formation (in two of the three siID1 groups). Similarly, and with improved consistency, the BxPC‐3 cells responded to *ID1* knockdown with a 50–80% reduction in protein levels and a corresponding 50–85% reduction in VM‐like structures (Fig. 2I–L). Figure 2C,G,K also illustrate the variation in VM capability by different human cancer cell lines, with noticeable distinction in the cellular composition of the VM structures formed and their number per area.

### 3.3 Inhibiting ID1 function via the small molecule inhibitor AGX‐51

**Fig. 3:**
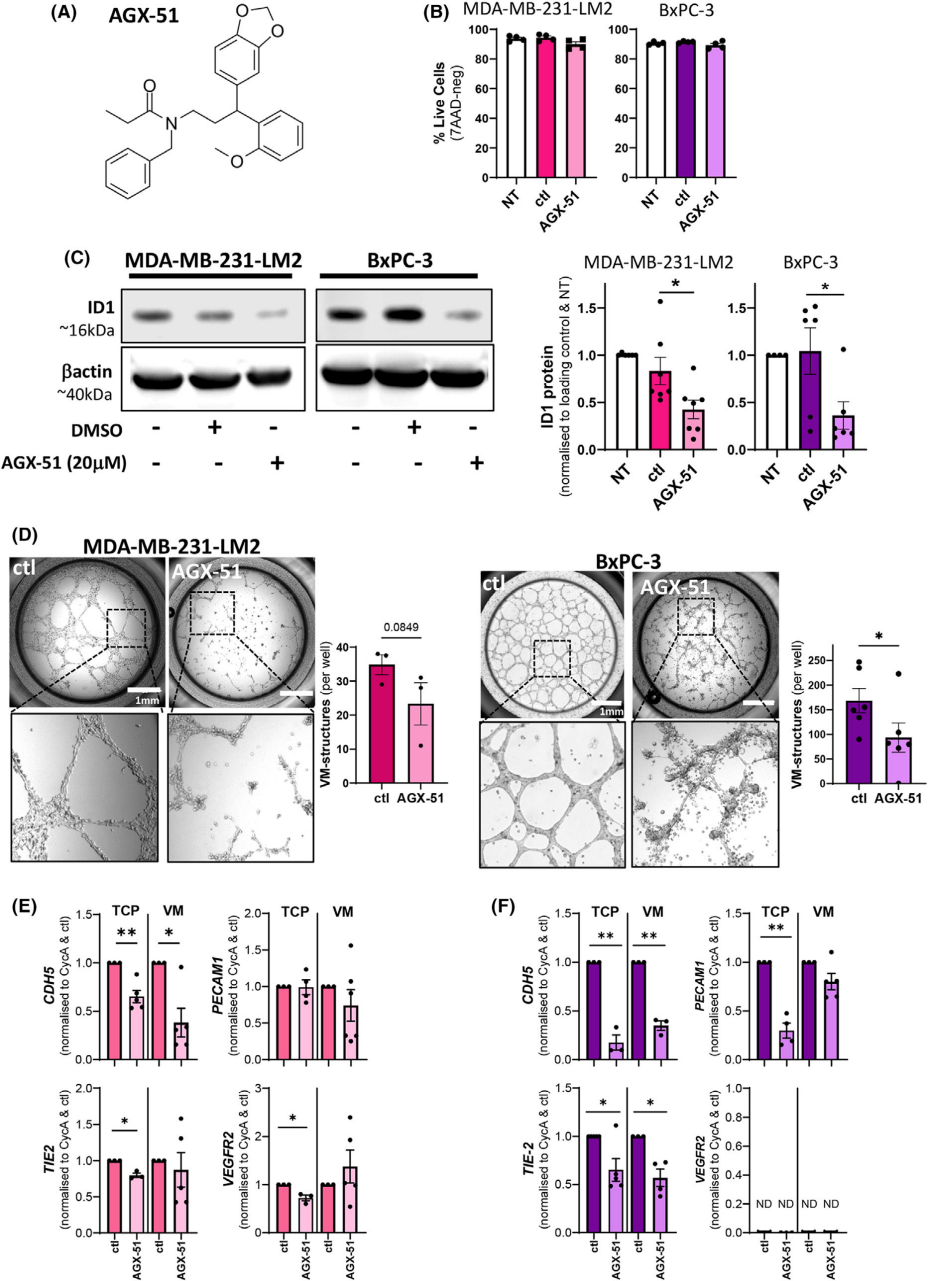
ID1 inhibition via small molecule inhibitor attenuates VM by cancer cells *in vitro*. (A) Chemical structure of AGX‐51. (B) Cell survival of MDA‐MB‐231‐LM2 and BxPC‐3 cancer cells treated with 20 μm AGX‐51 or DMSO (vehicle control, ctl) for 24 h as determined by 7‐AAD‐negative staining using flow cytometric analysis. Data expressed as mean ± SEM; *n* = 4 independent experiments. (C) Representative immunoblots of ID1 protein expression in MDA‐MB‐231‐LM2 and BxPC‐3 cancer cells treated with 20 μm AGX‐51 or DMSO diluent for 24 h. Quantified densitometry of immunoblots in bar graphs with data expressed as mean ± SEM from *n* = 7 (MDA‐MB‐231‐LM2; 6 independent experiments), *n* = 6 (BxPC‐3; 4 independent experiments) experiments. *P < 0.05 vs ctl, one‐way ANOVA. (D) Representative images of MDA‐MB‐231‐LM2 and BxPC‐3 cancer cells treated with 20 μm AGX‐51 or DMSO diluent for 24 h and quantitation of VM formation by cancer cells 24 h following seeding into Matrigel. Data are expressed as mean ± SEM from *n* = 3 (MDA‐MB‐231‐LM2) or *n* = 6 (BxPC‐3) individual experiments. *P < 0.05 vs DMSO, one‐way ANOVA. (E, F) Expression of VM signature genes *CDH5* (*VE‐Cadherin*), *PECAM1* (*CD31*), *TIE2*, and *VEGFR2* for MDA‐MB‐231‐LM2 (E) and BxPC‐3 (F) cancer cells pre‐treated ±20 μm AGX‐51 (or equivalent DMSO control (ctl)) for 24 h on TCP (2D) or in VM formation (3D) on Matrigel. ND = not detected. Data are expressed as mean ± SEM; with 3–6 replicates from three independent experiments. *P < 0.05, **P < 0.01 vs DMSO control, Welch's unpaired *t*‐test.

To further investigate a role for ID1 in VM formation by cancer cells, the first‐in‐class pan‐ID antagonist and degrader AGX‐51 was used (Fig. 3A) [37]. Exposure of the MDA‐MB‐231‐LM2 and BxPC‐3 cells to 20 μm of AGX‐51 (or diluent DMSO as control) for 24 h did not compromise cell survival (Fig. 3B) but significantly reduced ID1 protein expression by > 50% (Fig. 3C). To assess the ability of AGX‐51 to inhibit VM formation by the cancer cells, we exposed the MDA‐MB‐231‐LM2 and BxPC‐3 cells to 20 μm of AGX‐51 (or diluent) prior to seeding into the VM assay for a further 24 h prior to analysis. Figure 3D shows a modest, non‐significant reduction in VM structures by the MDA‐MB‐231‐LM2 cells and a more robust reduction in VM structures by the BxPC‐3 cells. To examine whether renowned vascular genes (e.g. VE‐cadherin (*CDH5*), *PECAM1* (CD31), *Tie‐2* and *VEGFR2*) were expressed by the cancer cells and, moreover, altered following exposure to AGX‐51 during VM formation on Matrigel, gene expression analysis was performed. Figure 3E,F show that AGX‐51 significantly reduces VE‐cadherin (*CDH5*) expression in MDA‐MB‐231‐LM2 and BxPC‐3 cells, both on TCP (2D) and VM (3D) formation. AGX‐51‐induced reduction of *PECAM‐1*, *TIE2*, and *VEGFR2* was observed more consistently in the TCP cultured cells. Notably, *VEGFR2* was not detectable in the BxPC‐3 cells (Fig. 3F).

### 3.4 Investigating the mechanism by which ID1 regulates VM formation

**Fig. 4:**
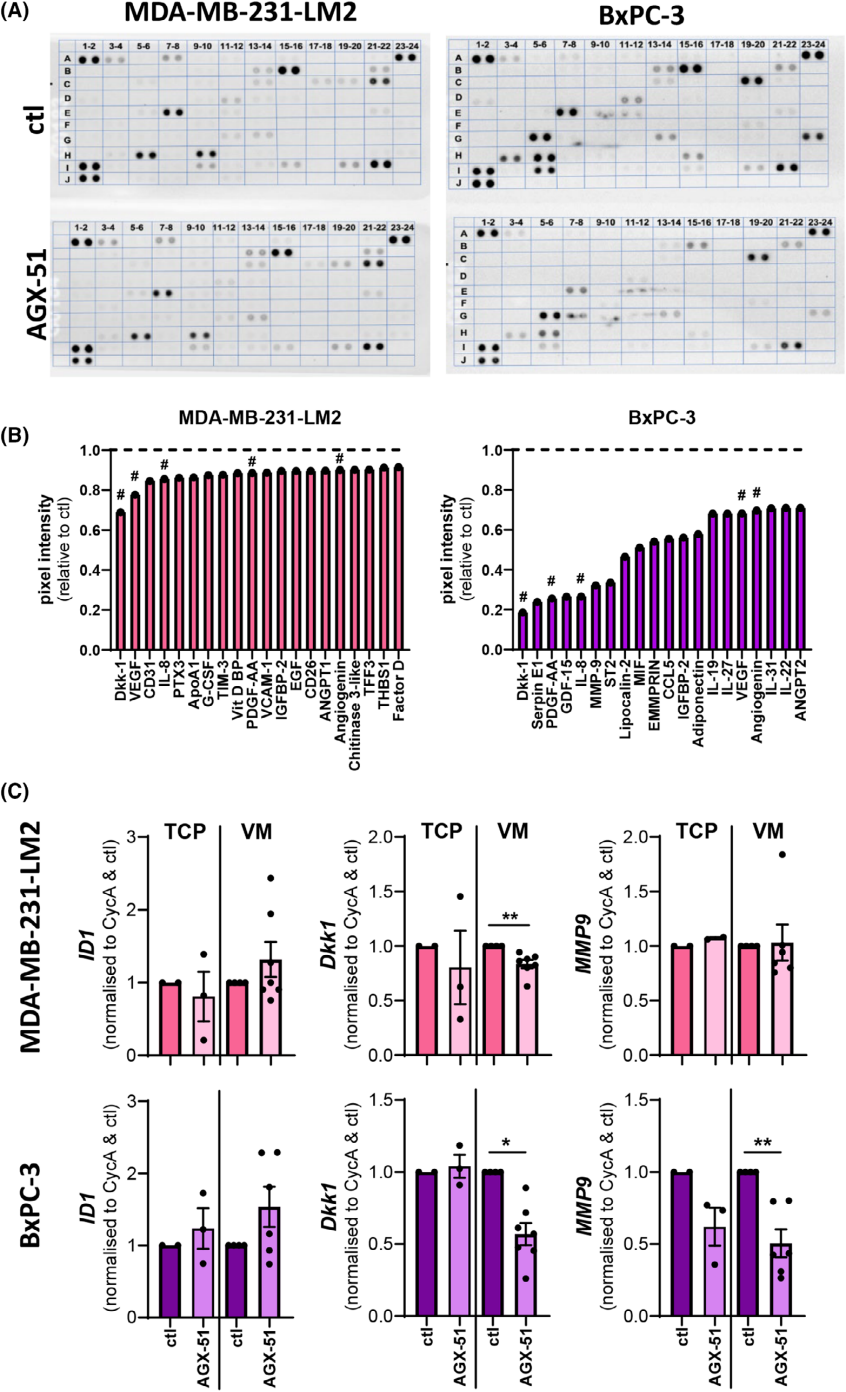
ID1 inhibition alters cancer cell secretome. (A) Protein profiler array immunoblots of secreted proteins in cell culture supernatant of MDA‐MB‐231‐LM2 and BxPC‐3 cancer cells pre‐treated with or without 20 μm AGX‐51 for 24 h. (B) Quantification of pixel intensity of duplicate dots via densitometry with the top 20 differentially expressed proteins shown in a bar graph as normalised to vehicle control (DMSO – dashed line) samples following subtraction of background for *n* = 1 experiment. ^#^Same proteins regulated across the two cancer cell types. (C) Gene expression of *ID1*, *DKK1*, and *MMP9* for MDA‐MB‐231‐LM2 and BxPC‐3 cancer cells pre‐treated with or without 20 μm AGX‐51 for 24 h on 2D tissue culture plastic (TCP) or 3D VM formation in Matrigel. Data are expressed as mean ± SEM from 4 independent experiments, with 4–7 replicates. *P < 0.05, **P < 0.01 vs DMSO control, Student paired *t*‐test.

A large body of research has identified ID1 to be involved in cancer progression with roles in cellular transformation, proliferation, survival, colony formation, invasion, metastasis, tumour angiogenesis by ECs and chemoresistance (reviewed in Ref. [19]). There is also emerging evidence for ID1 to mediate VM formation in hepatocellular carcinoma [38]. To further examine how ID1 might regulate VM formation, we utilised a cytokine protein array to identify differences in the secretome of AGX‐51‐treated MDA‐MB‐231‐LM2 and BxPC‐3 cells compared to control (DMSO) treated cells. Figure 4A illustrates the probed membranes identifying up to 105 proteins from the culture supernatants with densitometry quantifying comparative levels. The top 20 differentially regulated proteins for the two cell lines are shown in Fig. 4B, with MDA‐MB‐231‐LM2 cells showing decreases in proteins such as Dickkopf‐1 (DKK‐1), VEGF, CD31, IL‐8, platelet‐derived growth factor (PDGF)‐AA, and angiogenin. These six proteins were also lower in the secretome of the AGX‐51‐treated BxPC‐3 cells, with additional suppression of proteins Serpin E1, matrix metalloproteinase 9 (MMP9), extracellular matrix metalloproteinase inducer (EMMPRIN), and CCL5. The complete list of array data is presented in Table S1. As expected, BxPC‐3 cells (which express higher endogenous levels of ID1 protein (Figs 2 and 3)) exhibited a more profound response to ID1 inhibition by AGX‐51 than the MDA‐MB‐231‐LM2 cells.

Results from the cytokine array were further examined for changes in gene expression in cells cultured as 2D on TCP or 3D in VM Matrigel assays. Notably, *ID1* gene expression did not alter in response to AGX‐51 treatment in either MDA‐MB‐231‐LM2 or BxPC‐3 cells (Fig. 4C); this supports the documented mode of action of AGX‐51 for ubiquitin‐mediated degradation of ID1 protein [37]. In contrast, expression of *DKK‐1* was significantly reduced following the loss of ID1 in VM cultures of MDA‐MB‐231‐LM2 and BxPC‐3 cells. The BxPC‐3 cells (in VM formation) also exhibited a significant reduction in *MMP9* gene expression following AGX‐51 treatment.

### 3.5 E‐box motifs and regulation of gene expression by ID1

**Fig. 5:**
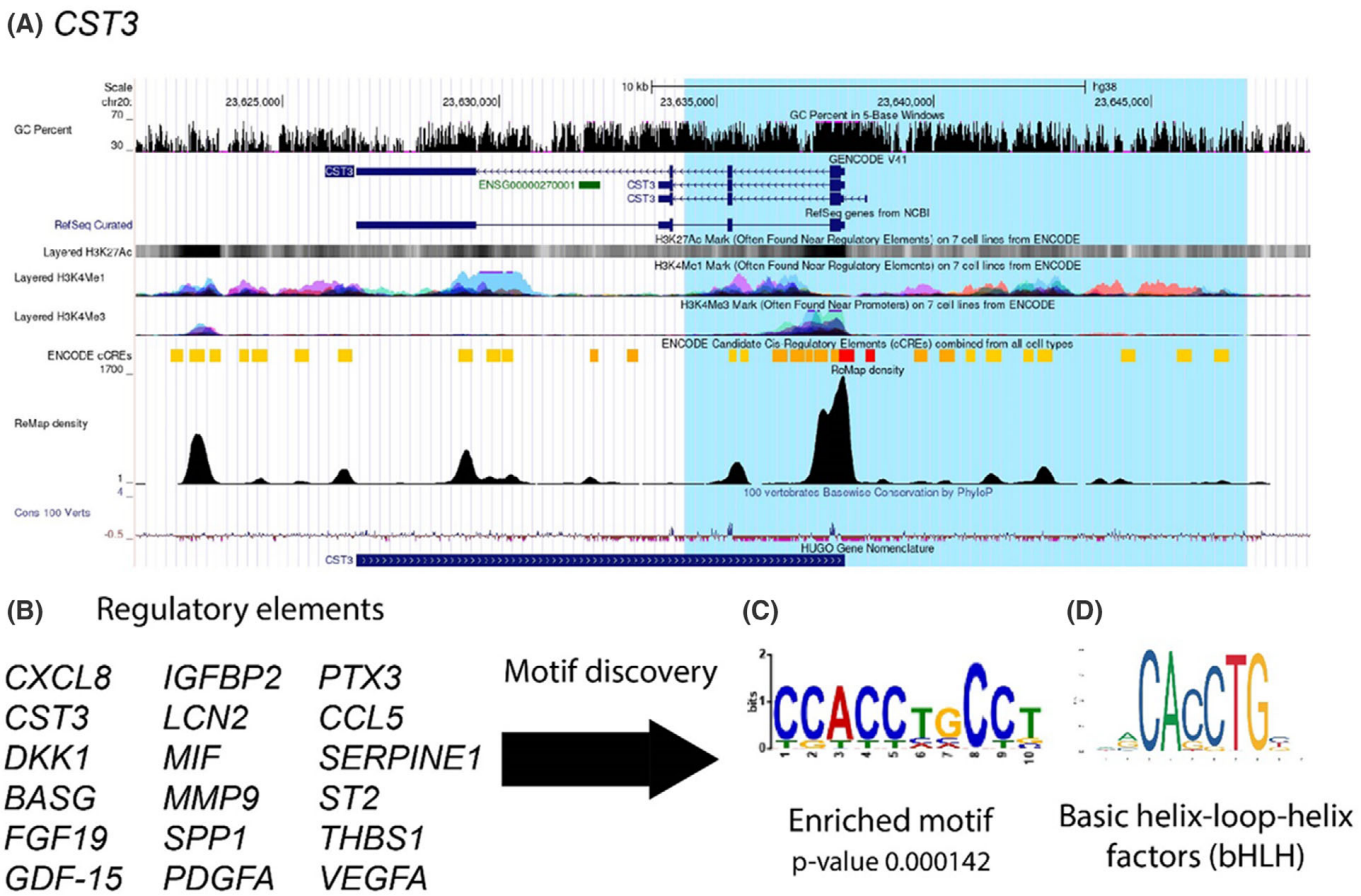
Motif discovery within cis‐regulatory elements of ID1 responder genes. (A) Screenshot of the UCSC Genome Browser (v438, hg38) of the selected region (blue highlight) of *CST3* based on the histone modifications H3K27Ac (“Layered H3K27Ac”), H3K4Me1 (“Layered H3K4Me1”) and H3K4Me3 (“Layered H3K4Me3”), the ENCODE Candidate Cis‐Regulatory Elements (“ENCODE cCREs”), and conservation of 100 vertebrate species (“Cons 100 Verts”). (B) Motif discovery was conducted using the sequences of putative cis‐regulatory elements of these 18 downregulated genes. (C) CANNTG was identified as a significantly enriched motif, the motif of basic helix–loop–helix factors (D).

Sequence homology between the ID family of proteins and the helix–loop–helix (HLH) proteins (primarily the group of E proteins; e.g. E47) facilitates a heterotypic interaction. When dimerised to IDs, the HLH proteins are rendered non‐functional as they are unable to attach to the E‐box motif (CANNTG) necessary for DNA binding. By sequestering bHLH proteins away from the DNA, ID1 directly modulates gene transcription. In the absence of ID1, bHLH proteins dimerise and bind DNA, which then facilitates gene transcription (reviewed in Ref. [39]). The extent of gene regulation by ID1 in cancerous cells is yet to be fully realised. To address this, for candidate genes identified in the Protein Profiler Array (Fig. 4), enriched motifs for candidate *cis*‐regulatory elements (cCRE) were selected based on H3K27Ac (open chromatin), H3K4me1 (enhancer) and H3K4me3 (promoter) histone modifications [29, 30, 31]. Figure 5A exemplifies the analysis process using the *CST3* gene with a screenshot of the UCSC Genome Browser of the selected region based on the histone modifications, the enriched cCRE motifs, and the species conservations. From a selection of 18 proteins implicated in regulation by ID1, all revealed an enrichment of E‐box motifs in their regulatory elements (Fig. 5B,C). Figure 5D illustrates the compatibility of our identified sequence to the highly conserved bHLH motif.

### 3.6 *ID1* expression in metastasis and gene ontology profiling

**Fig. 6:**
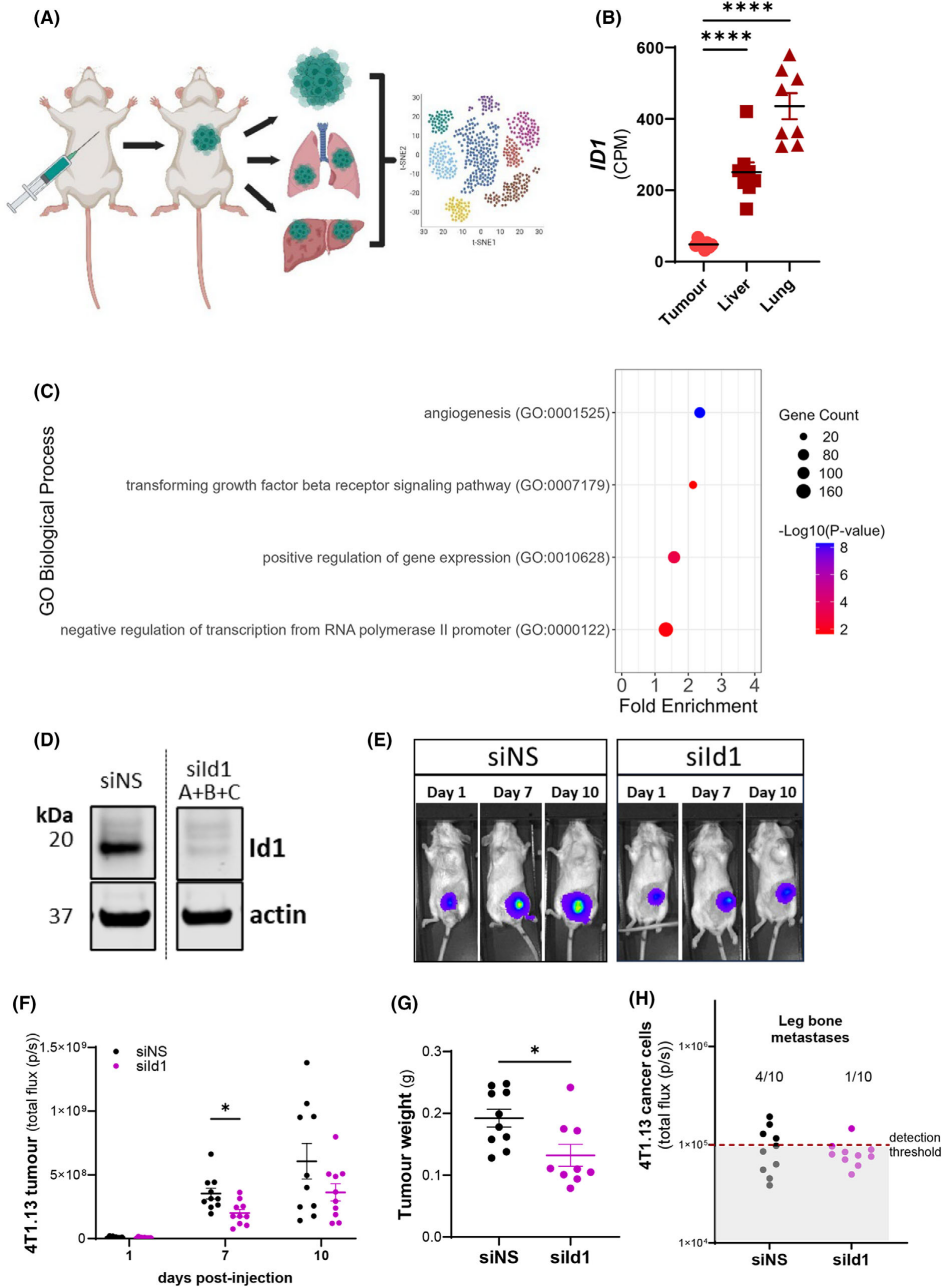
ID1 expression in the primary and metastatic MDA‐MB‐231 tumours and gene ontology analysis. (A) Schematic of the mouse xenograft bulk RNA‐sequencing data of LeGO‐barcoded MDA‐MB‐231 subclone cells from primary tumours and metastatic sites (liver and lung) in NOD scid gamma (NSG) mice available via GSE193369 with the tSNE plot clusters used for illustration purposes only. (B) Relative quantification of *ID1* gene levels in the MDA‐MB‐231 primary tumour, liver, and lung metastases from eight individual mice. Means ± SEM are shown; ****P < 0.0001 vs tumour, one‐way ANOVA. (C) Dot plot of enriched GO pathways in differentially expressed genes identified between primary tumours versus combined liver and lung metastatic tumours, showing results with a ≤ 0.05 false discovery rate (FDR). Fold enrichment is indicated on the x‐axis, with legend colours indicating the −log_10_ Benjamini‐Hochberg adjusted *P*‐values and the dot size proportional to the number of differentially expressed genes (DEG) in the given pathway. (D) Representative western blot of siRNA knockdown of *Id1* in 4T1.13‐LucGFP cells, where cells were treated with 50 nm non‐targeting siRNA (siNS), or a combination of three *Id1* targeting‐siRNA oligo duplexes ((si*Id1* A + B + C) combined total of 50 nm), total actin used as a loading control (representative of 3 independent experiments). 5 × 10^5^ 4T1.13‐LucGFP cells were engrafted into the fourth mammary fat pad of 6–8 week old female BALB/c mice, and tumours were harvested at day 10 post‐injection. 4T1.13‐LucGFP cells were pre‐treated ± si*Id1* for 72 h prior to being injected. (E) Representative bioluminescence images of tumour bearing mice at 1, 7, and 10 days post cancer cell injection. (F) Compiled luminescence data (total flux; photons·s^−1^ (p·s^−1^)) performed at days 1, 7, and 10. (G) Excised tumour weights (grams (g)). (H) Metastasis quantification in excised leg bones at the experimental endpoint (day 10), where signal is above 1 × 10^5^ photons/s. All data expressed as mean ± SEM, *n* = 10 mice per group; *P < 0.05, one‐way ANOVA with Dunnett's multiple comparisons test.

With our *in vitro* assays demonstrating that high expression of ID1 promotes VM, we next investigated if *ID1* expression varied during cancer progression. To address this, we performed *in silico* analysis of existing bulk RNA‐sequencing data of LeGO‐barcoded MDA‐MB‐231 subclones in primary and metastatic sites (primary tumour, liver and lung metastases) in NSG mice (GSE193369) [33]. The schematic in Fig. 6A shows the predominant organs for MDA‐MB‐231 cells metastasising from the primary tumour, where they exist as several subclones, represented by the t‐SNE cell clusters. Figure 6B shows that when MDA‐MB‐231 cells metastasise from the primary tumour to either the liver or the lung, *ID1* expression is significantly elevated in the distant tumours, as shown by increased *ID1* counts per million (CPM). A comparative analysis of *ID1*, *ID2* and *ID3* expression further implicated *ID1* as the dominant family member during disease progression with a 10‐fold higher expression in the primary tumour that increased further during metastasis (Fig. S2).

To better understand how *ID1* might relate to biological processes during breast cancer progression, we performed differential gene expression (DGE) analysis between primary tumours versus combined liver and lung samples using degust [34]. The resulting differentially expressed genes were analysed via gene ontology (GO) enrichment analysis to identify pathways enriched in *ID1* high expressing liver and lung tumours. The GO enrichment results in Fig. 6C (FDR ≤ 0.05, Benjamini‐Hochberg adjusted *P*‐values) indicate that when *ID1* is elevated, there is a significant enrichment in genes associated with ‘angiogenesis’, ‘transforming growth factor beta signalling pathway’ and global positive and negative regulation of gene regulation and transcription.

The resulting GO pathway genes identified in Fig. 6C were analysed for the occurrence of regulatory element containing genes in Fig. 5B, which returned four matches. *CXCL8* was identified in both the ‘angiogenesis’ (GO:0001525) and positive regulation of gene expression (GO:0010628) pathways. *PDGFA* and *SERPINE1* occurred in the ‘angiogenesis’ (GO:0001525) pathway and *DKK1* was identified in both the ‘positive regulation of gene expression’ (GO:0010628) and ‘negative regulation of transcription from RNA polymerase II promoter’ (GO:0000122) pathways. Taken together, these results support our contention that ID1 facilitates VM by cancer cells to assist disease progression.

To directly address whether manipulation of *Id1* could alter breast cancer progression, *Id1* was transiently knocked down in 4T1.13‐LucGFP mouse breast cancer cells via a combination of three *Id1*‐targeting siRNA duplexes (Fig. 6D). 4T1.13‐LucGFP cells (treated with non‐silencing or *Id1*‐targeting siRNAs for 72 h prior to injection into the mammary fat pad of Balb/c mice) were monitored for disease progression using bioluminescence imaging over 10 days post‐engraftment (Fig. 6E). Figure 6F shows that tumour bioluminescence (flux) was significantly reduced in the *Id1* knockdown group at 7 days post‐engraftment. A significant reduction in harvested tumour weights was also observed at the experimental endpoint (Fig. 6G). Tissues were harvested to scan for the presence of cancer cell metastasis, and while no cancer cells were detected in the liver or lungs of these mice (at experimental end point of day 10), a signal was detected in some of the long bones (as is expected with the 4T1.13 cells that are highly metastatic to the bone [40]). More specifically, bioluminescence was detected in 4 out of 10 mice of the control group, but only 1 out of 10 of the *Id1* knockdown group (Fig. 6H).

## 4 Discussion

To adapt to their microenvironment and survive, cancer cells rely on an intrinsic plasticity in their genome to orchestrate fundamental changes to their gene expression profile and morphology. Understanding the biological processes that modulate cancer cell adaptation is critical for uncovering vulnerabilities that are targetable. Of particular interest are the cancerous cells that are capable of forming vascular‐like structures. The presence of VM in tumours is associated with poor prognosis for many cancer patients, including those with cancer of the larynx, ovary, breast, skin, cervix, oesophagus, lung, stomach, intestine, brain, kidney, pancreas and more [7, 8, 9]. Hendrix and colleagues pioneered the VM field by first showing that melanoma cells could form tubular structures that were lumen‐containing and fluid‐conducting [6]. While VM by cancer cells has been controversial, the formation of fluid‐conducting structures by non‐ECs has also been documented for tumour‐associated macrophages *in vivo* [41] as well as trophoblasts in the uterine wall, which form vascular‐like structures to facilitate the blood supply during placental development [42, 43]. Herein, we present new information on transcriptional events that occur ‘early’ in VM formation by cancer cells.

Although most, if not all, cancer cells within a tumour will share the same driver mutations that provide a survival advantage, the tumour microenvironment also strongly influences the fitness of individual subclones [44]. To this end, cancer cells can respond to their surroundings by activating a cascade of events that promote VM formation. Our results suggest that within a few hours of placement into the ‘cancerous’ environment that is rich in pro‐angiogenic cues, the genomic transcript of the cancer cells begins to change. More specifically, within 6 h of placement onto Matrigel, MDA‐MB‐231‐LM2 TNBC cancer cells respond by rapidly upregulating transcripts that influence gene transcription (*ID1, ETV1, KLF4, TSEN15, ID3*), cell survival (*GRB10, MET*), cell cycling (*CDKN1B, CCNG, MIR100HG*), signalling pathways (*SPRED1, SPRY1, SMAD7*), intracellular transport (*SYPL1, TMED1*), and cell adhesion (*ITGA2, CLDN12*). Most notably, many of these early response alterations in the cancer cells are known pro‐angiogenic transcripts, including *ITGA2* [45], *CLDN12* [46], *SMAD7* [47], *MET* [48], *KLF4* [49, 50], *ID3* [51] and *ID1* [51].

Amongst this list, the ‘inhibitor of DNA binding’ (*ID*) family of genes were of particular interest because of their broad influence on gene regulation. This family of four genes (*ID1‐4*) are members of the helix–loop–helix (HLH) proteins that form heterodimers with basic HLH proteins [52]. IDs have no DNA binding capability *per se* but prevent bHLH proteins from binding to their cognate E‐box motifs in the DNA, thus causing changes in transcriptional activity [20]. By interfering with gene transcription events, IDs regulate many biological processes including cell differentiation, proliferation, migration, angiogenesis during embryonic development [51], as well as tumour vasculature [51, 53], EMT and metastasis [19]. While our data suggest that both *ID1* and *ID3* increase ~ 3‐fold within the first 6 h of VM formation by TNBC (and both are pro‐angiogenic [51, 54]), *ID1* expression was significantly higher (5–10 fold) than *ID3* across all culture conditions, thus warranting further investigation. Notably, low level *ID2* was also detected, but expression did not change during VM formation. A role for *ID1* in VM formation by TNBC (MDA‐MB‐231‐LM2 cells) and PDAC (BxPC‐3 and AsPC‐1) cancer cells was supported via *ID1*‐targeting siRNA knockdown; and concurs with a recent study by Zhang and colleagues on hepatocellular carcinoma cells [38]. Our inhibition of VM formation was most evident in the BxPC‐3 PDAC cells which contain the highest baseline expression of ID1 protein. Consistent with this, inhibition of VM was also observed with the addition of the pan‐ID inhibitor AGX‐51 that destabilises ID proteins from binding to the E‐box of bHLH proteins, marking the IDs for ubiquitin‐mediated degradation (without significantly altering *ID1* gene expression) and releasing the E‐box proteins to assist gene transcription events [37]. Interestingly, exposure of VM‐competent cancer cells to AGX‐51 also caused a reduction in highly regarded vascular genes, e.g., VE‐cadherin and Tie2.

To investigate the regulatory factors influenced by ID1 during VM formation, an analysis of cancer cell secretome was performed using the culture supernatants from VM forming MDA‐MB‐231‐LM2 and BxPC‐3 cells treated with and without AGX‐51. Our data show that the BxPC‐3 cells exhibited the best response to AGX‐51 treatment with a 30–80% reduction in 20 secreted proteins. Notably, proteins such as DKK‐1, VEGF, IL‐8, PDGF‐AA, and angiogenin were downregulated within both the PDAC and TNBC cancer cells. Transcriptional control of these proteins by ID1/bHLH proteins is further supported by the identification of E‐box motifs (CANNTG) in their regulatory elements. For example, an E‐box motif was identified for Dickkopf‐1 (DKK‐1), an inhibitor of the Wnt/β‐catenin signalling pathway [55] that is elevated in advanced breast and pancreatic cancer patients [56] and is known to promote immune evasion [55, 57]. DKK‐1 activates the Akt pathway to promote tumour progression, cell survival, and protease production for cancer cell migration [56, 58]. To the best of our knowledge, a role for DKK‐1 in VM has not been documented. It is our contention that ID1 is a strong regulator of DKK‐1 in cancer cells and implicates known VM pathways (P13K/AKT and ERK) to promote extracellular matrix (ECM) remodelling and ultimately VM formation.

Furthermore, administration of AGX‐51 reduced the availability of VEGF, the leading angiogenic protein essential for neovascularisation and vascular permeability [59, 60], documented to promote VM by cancer cells [61] and a known regulatory target of ID1 [62]. Further support for ID1 in VM formation includes the modulation of PDGF, a known regulator of VEGF expression in cancer [63] and a modulator of cellular transformation [64]. Our results also implicate ID1 regulation of SERPINE1 (plasminogen activator inhibitor‐1 (PAI‐1)) which is reported to promote tumour vasculature by protecting ECs from apoptosis [65], and is associated with poor prognosis in cancer patients [66]. We believe this to be the first report linking *SERPINE1* to ID1 and VM, and propose that ID1 upregulates *SERPINE1* to produce serine protease inhibitors to control blood clotting, a crucial step in the generation of fluid‐conducting VM structures [11]. Results herein support existing data of ID1 regulating IL‐8 [67], a pro‐inflammatory cytokine that is constitutively expressed by many tumour cells to attract and modulate neutrophils, mediate cell metastasis, enhance vascular permeability, and promote VM formation [68, 69]. Our observation of ID1 regulating MMP9 secretion is of interest in cancer progression as it degrades the extracellular matrix and basement membrane components to promote cancer metastasis [64]. MMP9 also liberates locally trapped VEGF to further promote angiogenesis [70] and is a known mediator of VM [16, 17].

Having acquired *in vitro* evidence that the local environment significantly impacts the transcriptomic profile of cancer cells, we turned to single‐cell tracking methods as a powerful tool to better understand the complexity of cancer metastasis *in vivo*. Moreover, we utilised an optical barcoding study that tracked individual MDA‐MB‐231 cancer cell subclones [33] to evaluate *ID1* expression. Our results suggest the ID1 pathway is upregulated in TNBC metastasis to the liver and even higher in metastasis to the lung when compared to primary tumours. While our data imply a dominant role for ID1 over ID2 and ID3, purely based on the 5–10‐fold higher expression of *ID1* in primary, liver, and lung cancer samples, a modest increase in *ID2* and *ID3* was also observed between the primary tumour and lung metastases. With the relative *ID1* expression patterns in the primary tumour, lung and liver metastases being highly reproducible between mice, these processes are not random but are likely to be dictated by the tumour microenvironment. This concept is supported by an earlier publication by Minn and colleagues who listed *ID1* as a transcriptional regulator of the lung metastasis gene signature of human primary breast tumours as well as MDA‐MB‐231 cells injected orthotopically into the mammary fat pad of mice [18]. Notably, the Minn publication did not mention tumour vasculature or VM. Our observation of elevated *ID1* in TNBC metastases *in vivo*, together with gene ontology profiling for transcriptional regulation of a vascular‐like phenotype by the cancer cells, provides the strongest evidence to date that *ID1* is a higher‐order regulator of VM by cancer cells. A better understanding of this regulatory control may lead to a tumour‐specific vulnerability amenable to therapeutic interference in TNBC as well as other cancers known to express high levels of *ID1*, including pancreatic (shown here), bladder [71], brain [72], head and neck [73], ovarian [74] and more (reviewed in Ref. [39]).

## 5 Conclusion

For over three decades, the ID proteins have been of clinical interest as functional regulators of the bHLH transcription factors that facilitate cancer cell proliferation and multipotency [39]. Our observations further implicate ID1 in cancer progression as a master regulator of VM by cancer cells and mediator of lung and liver metastases. With building evidence that elevated levels of ID1 in cancer cells also contribute to chemotherapy [75] and radiotherapy resistance [76, 77], controlling ID1 expression and/or function warrants further investigation. The small molecule pan‐ID inhibitor AGX‐51 begins to address this opportunity, but with poor solubility and lack of ID1 specificity, improvements are recommended. In another approach, Henke and coworkers developed an anti‐tumour agent that downregulates ID1 effectively in tumour ECs, which yielded a significant reduction in breast cancer growth and metastasis [78]. Our work supports further development of anti‐ID1 reagents with the proposed impact directly on cancer cells for inhibition of VM and reduction of disease progression.

## Conflict of interest

The authors declare no conflict of interest.

## Author contributions

EJT, ELD, KJ, NN, MT, KKMM, MPC, AF, JT and MD performed the experiments. EJT, ELD, KJ, NN, JT, MD, and CSB performed the analysis. DM provided unique resources and advice. EJT and CSB wrote the first draft of the manuscript. All authors contributed to the manuscript revision, read, and approved the submitted version.

## Peer review

The peer review history for this article is available at https://www.webofscience.com/api/gateway/wos/peer‐review/10.1002/1878‐0261.70027.

## Supporting information


**Fig. S1.** *ID1* expression in MDA‐MB‐231‐LM2 cells under hypoxia.
**Fig. S2**. *ID1‐3* expression in MDA‐MB‐231 cells in mice.
**Table S1**. Complete BxPC3 and MDA‐MB‐231‐LM2 cell secretomes from protein profiler arrays.

**Video S1.** MDA‐MB‐231‐LM2 cells in Matrigel undergo vasculogenic mimicry.

## Data Availability

The data supporting the findings of this study are available within the article and its Supporting Information. Transcriptomic raw data have been deposited into the publicly available Gene Expression Omnibus (GEO) with the accession number GSE272838. Data analysed in Fig. 6 (and Fig. S2) were obtained from GEO with the accession number GSE193369.
