# Supplementary material for: Inhibitor of DNA binding‐1 is a key regulator of cancer cell vasculogenic mimicry
Source: Mol Oncol. 2025 Mar 21;19(9):2537–56. doi: 10.1002/1878-0261.70027 (PMC12420356; doi:10.1002/1878-0261.70027)
Supplement: Supplementary file 1 — Fig. S1. ID1 expression in MDA‐MB‐231‐LM2 cells under hypoxia. Fig. S2. ID1‐3 expression in MDA‐MB‐231 cells in mice. Table S1. Complete BxPC3 and MDA‐MB‐231‐LM2 cell secretomes from protein profiler arrays. [file MOL2-19-2537-s001.pdf]

Inhibitor of DNA binding-1 is a key regulator of cancer cell vasculogenic mimicry

Emma J. Thompson[1], Emma L. Dorward[1], Kristyn Jurrius[1], Nathalie Nataren[1], Markus Tondl[1], Kay K. Myo Min[1], Michaelia P. Cockshell[1], Anahita Fouladzadeh[1], John Toubia[1,2], Mark DeNichilo[1], Delphine Merino[3,4,5,6], and Claudine S. Bonder[1,7]

[1] Centre for Cancer Biology, University of South Australia and SA Pathology, Adelaide, SA, Australia
[2] ACRF Cancer Genomics Facility, Centre for Cancer Biology, University of South Australia and SA Pathology, Adelaide, SA, Australia
[3] Olivia Newton John Cancer Research Institute, Melbourne, VIC, Australia
[4] School of Cancer Medicine, La Trobe University, Bundoora, VIC, Australia
[5] Department of Medical Biology, The Faculty of Medicine, Dentistry and Health Science, The University of Melbourne, Melbourne, VIC, Australia
[6] Immunology Division, The Walter and Eliza Hall Institute of Medical Research, Parkville, VIC, Australia
[7] Adelaide Medical School, University of Adelaide, Adelaide, SA, Australia

Supplementary Video 1. MDA-MB-231-LM2 cells in Matrigel undergo vasculogenic mimicry. 1.75-3.5x10[4] cancer cells were seeded onto a layer of Growth Factor Reduced Matrigel and images captured over 24 hours using disk confocal live microscopy (CV100, Olympus, Tokyo, Japan).

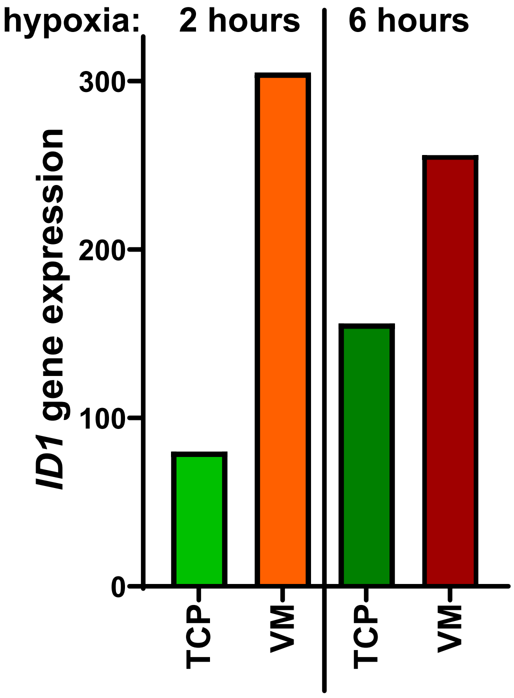

Supplementary Figure S1. ***ID1*** **expression in MDA-MB-231-LM2 cells under hypoxia**
Relative gene expression of *ID1* in MDA-MB-231-LM2 cells grown on tissue culture plastic (TCP) versus a vasculogenic mimicry (VM) assay at 2 hour and 6 hour time points under hypoxic conditions (0.5% O_2, 5% CO_2).

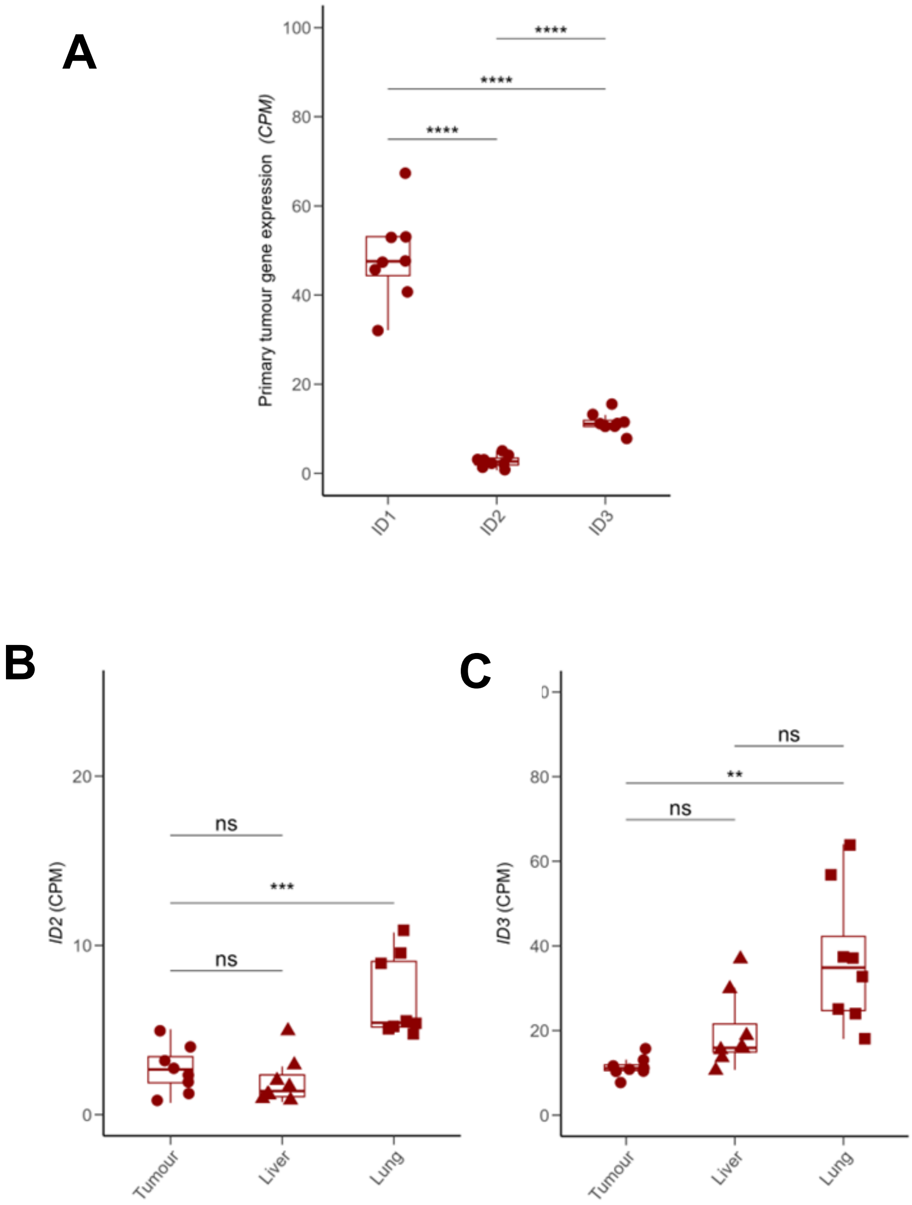

Supplementary Figure S2. ***ID1-3*** **expression in MDA-MB-231 cells in mice**
(A) Relative quantification of *ID1-3* gene levels in the MDA-MB-231 primary tumour, liver and lung metastases from eight individual mice. ****p<0.0001 vs ID1 in the primary tumour, ANOVA. **(B)** Relative quantification of *ID2* expression in the MDA-MB-231 primary tumour, liver and lung metastases. ***p<0.001 vs *ID2* in the primary tumour, ANOVA. **(C)** Relative quantification of *ID3* expression in the MDA-MB-231 primary tumour, liver and lung metastases. **p<0.01 vs *ID3* in the primary tumour, ANOVA. Means ± SEM are shown from eight individual mice.

Supplementary Table 1
BxPC-3 cell secretome

**Protein name – gene ID – other names**	**Expression levels following AGX-51 treatment** (Fold change)
Dkk-1 - 22943 - Dickkopf-1	0.186
Serpin E1 – 5054 - PAI-I, PAI-1, Nexin	0.239
PDGF-AA – 5154	0.256
GDF-15 – 9518 – MIC-1	0.265
IL-8 – 3576 – CXCL8	0.266
MMP-9 – 4318 – CLG4B, Gelatinase B	0.322
ST2 – 9173 – IL-1 R4, IL1RL1, ST2L	0.336
Lipocalin-2 – 3934 – NGAL, LCN2, Siderocalin	0.465
MIF – 4282	0.511
EMMPRIN – 682 – CD147, Basigin	0.541
RANTES – 6352 – CCL5	0.555
IGFBP-2 – 3485	0.561
Adiponectin – 9370 – Acrp30	0.579
IL-19 – 29949	0.681
IL-27 – 246778	0.681
VEGF – 7422 – BEGFA	0.682
Angiogenin – 283	0.697
IL-31 – 386653	0.708
IL-22 – 50616 – IL-TIF	0.710
Angiopoietin-2 – 285 – Ang-2, ANGPT2	0.710
FGF-19 – 9965	0.716
IL-32 – 9235	0.729
IL-4 – 3565	0.731
BDNF – 627 – Brain-derived Neurotrophic Factor	0.733
Kallikrein 3 – 354 – PSA, KLK3	0.740
GROα – 2919 – CXCL1, MSGA-α	0.744
IL-23 – 51561 – IL-23A, SGRF	0.748
Leptin – 3952 – OB	0.753
Angiopoietin-1 – 284 – Ang-1, ANGPT1	0.759
IL-18 – 10068	0.769
IL-24 – 11009 – C49A, FISP, MDA-7, MOB-5, ST16	0.770
BAFF – 10673 – BLyS, TNFSF13B	0.779
IL-11 – 3589	0.781
IP-10 – 3627 – CXCL10	0.784
C-Reactive Protein – 1401 – CRP	0.795
Thrombospondin-1 – 7057 – THBS1, TSP-1	0.798
IL-10 – 3586	0.800
IL-17A – 3605 – IL-17, CTLA8	0.805
CD40 ligand – 959 – CD40L, TNFSF5, CD154, TRAP	0.808
Complement Factor D – 1675 – Adipsin, CFD	0.820

PDGF-AB/BB – 5154/5155	0.821
IL-5 – 3567	0.824
Pentraxin 3 – 5806 – PTX3, TSG-14	0.826
IL-3 – 3562	0.832
MCP-3 – 6354 – CCL7, MARC	0.835
Apolipoprotein A-I – 335 – ApoA1	0.842
Flt-3 Ligand – 2323 – FLT3LG	0.843
FGF basic – 2247 – FGF-2	0.847
Chitinase 3-like 1 – 1116 – CHI3L1, YKL-40	0.854
Osteopontin – 6696 – OPN	0.856
MIP-3α 6364 CCL20, Exodus-1, LARC	0.865
Endoglin – 2022 – CD105, ENG	0.865
ENA-78 – 6374 – CXCL5	0.865
Growth Hormone – 2688 – GH, Somatotropin	0.866
ICAM-1 – 3383 – CD54	0.867
IL-1ra – 3557 – IL-1F3	0.869
MIG – 4283 – CXCL9	0.873
IL-33 – 90865 – C9orf26, DVS27, NF-HEV	0.873
Fas Ligand – 356 – TNFSF6, CD178, CD95L	0.878
GM-CSF – 1437 – CSF2	0.883
IL-12 p70 – 3593	0.894
Resistin – 56729 – ADSF, FIZZ3, RETN	0.905
IL-1α – 3552 – IL-1F1	0.906
I-TAC – 6373 – CXCL11, SCYB9B	0.914
IL-15 – 3600	0.917
HGF – 3082 – Scatter Factor, SF	0.918
MIP-1α/MIP-1β – 6348/6351 – CCL3/CCL4	0.922
Complement Component C5/C5a – 727 – C5/C5a	0.924
IL-13 – 3596	0.927
IL-2 – 3558	0.927
TfR – 7037 – CD71, TFR1, TFRC, TRFR	0.944
PF4 – 5196 – CXCL4	0.945
Cystatin C – 1471 – CST3, ARMD11	0.948
FGF-7 – 2252 – KGF	0.951
Vitamin D BP – 2638 – VDB, DBP, VDBP	0.955
MCP-1 – 6347 – CCL2, MCAF	0.957
TIM-3 – 84868 – HAVCR2	0.961
IGFBP-3 – 3486	0.962
IL-16 – 3603	0.968
DPPIV – 1803 – CD26, DPP4, Dipeptidyl-peptidase IV	0.971
IL-1β – 3553 – IL-1F2	0.974
Relaxin-2 – 6019 – RLN2, RLXH2	0.985
Cripto-1 – 6997 – Teratocarcinoma-derived Growth Factor	0.988
CD31 – 5175 – PECAM-1	0.994

IL-34 – 146433 – C16orf77	0.995
TARC – 6361 – CCL17	1.000
IFN-γ – 3458 – IFNG	1.006
CD14 – 929	1.015
TGF-α – 7039 – TGFA	1.015
M-CSF – 1435 – CSF1	1.036
TFF3 – 7033 – ITF, TFI	1.047
RBP-4 – 5950	1.051
RAGE – 177	1.091
VCAM-1 – 7412 – CD106	1.109
Myeloperoxidase – 4353 – MPO, Lactoperoxidase	1.132
MIP-3β – 6363 – CCL19, ELC	1.252
SHBG – 6462 – ABP	1.257
TNF-α – 7124 – TNFSF1A	1.349
IL-6 – 3569	1.431
EGF – 1950 – Epidermal Growth Factor	1.442
uPAR – 5329 – PLAUR	2.083
SDF-1α – 6387 – CXCL12, PBSF	2.315
LIF – 3976	2.416
G-CSF – 1440 – CSF3	2.550
CD30 – 943 – TNFRSF8	3.546

MDA-MB-231-LM2 cell secretome

**Protein name – gene ID – other names**	**Expression levels following AGX-51 treatment** (Fold change)
Dkk-1 – 22943 – Dickkopf-1	0.690
VEGF – 7422 – BEGFA	0.777
CD31 – 5175 – PECAM-1	0.846
IL-8 3576 – CXCL8	0.856
Pentraxin 3 – 5806 – PTX3, TSG-14	0.862
Apolipoprotein A-I – 335 – ApoA1	0.864
G-CSF – 1440 – CSF3	0.875
TIM-3 – 84868 – HAVCR2	0.877
Vitamin D BP – 2638 – VDB, DBP, VDBP	0.885
PDGF-AA – 5154	0.886
VCAM-1 – 7412 – CD106	0.888
IGFBP-2 – 3485	0.896
EGF – 1950 – Epidermal Growth Factor	0.896
DPPIV – 1803 – CD26, DPP4, Dipeptidyl-peptidase IV	0.896
Angiopoietin-1 – 284 – Ang-1, ANGPT1	0.898
Angiogenin – 283	0.901
Chitinase 3-like 1 – 1116 – CHI3L1, YKL-40	0.902
TFF3 – 7033 – ITF, TFI	0.903
Thrombospondin-1 – 7057 – THBS1, TSP-1	0.913
Complement Factor D – 1675 – Adipsin, CFD	0.915

Cripto-1 – 6997 – Teratocarcinoma-derived Growth Factor	0.915
Adiponectin – 9370 – Acrp30	0.918
C-Reactive Protein – 1401 – CRP	0.920
CD40 ligand – 959 – CD40L, TNFSF5, CD154, TRAP	0.923
BAFF – 10673 – BLyS, TNFSF13B	0.930
Flt-3 Ligand – 2323 – FLT3LG	0.935
Angiopoietin-2 – 285 – Ang-2, ANGPT2	0.937
uPAR – 5329 – PLAUR	0.943
BDNF – 627 – Brain-derived Neurotrophic Factor	0.944
FGF-7 – 2252 – KGF	0.951
Complement Component C5/C5a – 727 – C5/C5a	0.953
TARC – 6361 – CCL17	0.956
EMMPRIN – 682 – CD147, Basigin	0.957
Endoglin – 2022 – CD105, ENG	0.966
IGFBP-3 – 3486	0.966
TfR – 7037 – CD71, TFR1, TFRC, TRFR	0.967
FGF basic – 2247 – FGF-2	0.970
Fas Ligand – 356 – TNFSF6, CD178, CD95L	0.977
IL-1α – 3552 – IL-1F1	0.978
ENA-78 – 6374 – CXCL5	0.982
GROα – 2919 – CXCL1, MSGA-α	0.985
IFN-γ – 3458 – IFNG	0.987
TGF-α – 7039 – TGFA	0.988
CD14 – 929	0.992
Serpin E1 – 5054 – PAI-I, PAI-1, Nexin	0.996
CD30 – 943 – TNFRSF8	1.000
ST2 – 9173 – IL-1 R4, IL1RL1, ST2L	1.001
HGF – 3082 – Scatter Factor, SF	1.002
SDF-1α – 6387 – CXCL12, PBSF	1.003
FGF-19 – 9965	1.003
ICAM-1 – 3383 – CD54	1.007
IL-11 – 3589	1.013
IL-1β – 3553 – IL-1F2	1.014
Growth Hormone – 2688 – GH, Somatotropin	1.015
IL-13 – 3596	1.016
IL-3 – 3562	1.017
M-CSF – 1435 – CSF1	1.022
IL-12 p70 – 3593	1.024
IL-5 – 3567	1.026
IL-1ra – 3557 – IL-1F3	1.027
IL-15 – 3600	1.027
IL-31 – 386653	1.029
MCP-3 – 6354 – CCL7, MARC	1.031
IL-10 – 3586	1.031
Resistin – 56729 – ADSF, FIZZ3, RETN	1.037
IL-32 9235	1.037
IL-27 246778	1.038
IL-2 – 3558	1.039
IL-4 – 3565	1.039
IL-33 – 90865 – C9orf26, DVS27, NF-HEV	1.046

IL-16 – 3603	1.051
IL-18 Bpa – 10068	1.054
PF4 – 5196 – CXCL4	1.054
IL-6 – 3569	1.055
GM-CSF – 1437 – CSF2	1.056
IL-23 – 51561 – IL-23A, SGRF	1.058
TNF-α – 7124 – TNFSF1A	1.059
PDGF-AB/BB – 5154/5155	1.060
IL-24 – 11009 – C49A, FISP, MDA-7, MOB-5, ST16	1.062
RAGE – 177	1.068
MIG – 4283 – CXCL9	1.072
IL-19 – 29949	1.076
IL-34 – 146433 – C16orf77	1.078
RANTES – 6352 – CCL5	1.079
SHBG – 6462 – ABP	1.080
IL-22 – 50616 – IL-TIF	1.081
LIF – 3976	1.084
I-TAC – 6373 – CXCL11, SCYB9B	1.086
Cystatin C – 1471 – CST3, ARMD11	1.091
IP-10 – 3627 – CXCL10	1.092
Kallikrein 3 – 354 – PSA, KLK3	1.093
MIP-3β – 6363 – CCL19, ELC	1.093
IL-17A – 3605 – IL-17, CTLA8	1.097
MIP-1α/MIP-1β – 6348/6351 – CCL3/CCL4	1.101
Lipocalin-2 – 3934 – NGAL, LCN2, Siderocalin	1.102
GDF-15 – 9518 – MIC-1	1.102
MMP-9 – 4318 – CLG4B, Gelatinase B	1.102
MCP-1 – 6347 – CCL2, MCAF	1.107
Relaxin-2 – 6019 – RLN2, RLXH2	1.112
RBP-4 – 5950	1.118
MIP-3α – 6364 – CCL20, Exodus-1, LARC	1.119
Leptin – 3952 – OB	1.125
Myeloperoxidase – 4353 – MPO, Lactoperoxidase	1.163
Osteopontin – 6696 – OPN	1.170
MIF – 4282	1.173
